# Activation of the PDGFRα-Nrf2 pathway mediates impaired adipocyte differentiation in bone marrow mesenchymal stem cells lacking Nck1

**DOI:** 10.1186/s12964-019-0506-4

**Published:** 2020-02-14

**Authors:** Nida Haider, Louise Larose

**Affiliations:** Division of Experimental Medicine, Department of Medicine, McGill University and The Research Institute of McGill University Health Centre, Montreal, QC H4A 3J1 Canada

**Keywords:** Adipogenesis, Nck1, PDGFRα, Nrf2, Bone marrow Mesenchymal stem/stromal cells (BM-MSCs)

## Abstract

**Background:**

The limited options to treat obesity and its complications result from an incomplete understanding of the underlying molecular mechanisms regulating white adipose tissue development, including adipocyte hypertrophy (increase in size) and hyperplasia (increase in number through adipogenesis). We recently demonstrated that lack of the adaptor protein Nck1 in mice is associated with reduced adiposity and impaired adipocyte differentiation. In agreement, Nck1 depletion in 3 T3-L1 cells also attenuates adipocyte differentiation by enhancing PDGFRα activation and signaling. This is accompanied by higher expression of PDGF-A, a specific PDGFRα ligand, that may contribute to enhanced activation of PDGFRα signaling in the absence of Nck1 in white adipose tissue. However, whether Nck1 deficiency also impairs adipogenic differentiation in bone marrow still remains to be determined.

**Methods:**

To address this point, Nck1-deficient derived bone marrow mesenchymal stem/stromal cells (BM-MSCs) and C3H10T1/2 mesenchymal stem cells were differentiated into adipocytes in vitro*.* Genes and proteins expression in these cellular models were determined using qPCR and western blotting respectively. Pharmacological approaches were used to assess a role for Nrf2 in mediating Nck1 deficiency effect on mesenchymal stem cells adipocyte differentiation.

**Results:**

Nck1 deficiency in both BM-MSCs and C3H10T1/2 results in impaired adipocyte differentiation, accompanied by increased activation of the transcription factor Nrf2, as shown by increased mRNA levels of Nrf2 target genes, including PDGF-A. Using pharmacological activator and inhibitor of Nrf2, we further provide evidence that Nrf2 is an important player in PDGFRα signaling that mediates expression of PDGF-A and impaired adipogenesis in Nck1-deficient BM-MSCs and C3H10T1/2 cells.

**Conclusion:**

This study demonstrates that Nck1 deficiency in mesenchymal stem cells impairs adipogenesis through activation of the PDGFRα-Nrf2 anti-adipogenic signaling pathway.

Video Abstract.

## Plain English summary

Restricting the expansion of fat tissues would be a great option to prevent obesity and associated complications. However, incomplete understanding of the mechanisms controlling fat tissues development and expansion leads to limited treatment options. In a previous study, we showed that the deficiency of the small protein Nck1 in mice results in reduced body fat accumulation mediated by increased signaling from PDGFRα in cells responsible to generate fat cells in white adipose tissue (WAT). In this study, we demonstrate that Nck1 deficiency in bone marrow mesenchymal stem/stromal cells (BM-MSCs) and C3H10T1/2 mesenchymal stem cells also impairs the differentiation of adipocytes through activation of the PDGFRα-Nrf2 anti-adipogenic signaling pathway. Our study highlights Nrf2 as an important player in mediating PDGFRα signaling that limits the generation of fat cells upon Nck1 deficiency.

## Background

White adipose tissue (WAT) development involves adipocyte hypertrophy (increase in size) and hyperplasia (increase in number) [1]. During normal WAT development, adipocyte hyperplasia is associated with the differentiation of precursor cells into adipocytes through adipogenesis, a process mainly observed in childhood and adolescence, whereas, expansion of WAT in adults appears to be limited to adipocyte hypertrophy [2]. In contrast, obesity is associated with an excessive expansion of WAT resulting from abnormal increases in both adipocyte hypertrophy and hyperplasia [1, 3]. Although in the past years important progress has been made, the underlying molecular mechanisms regulating these processes are still not well understood; therefore, contributing to limited pharmacological options to prevent or treat obesity.

Recently, we reported that the Src homology (SH) domain-containing adaptor protein Nck1 is required for normal WAT development in mice. Indeed, we demonstrated that Nck1 deficiency in mice is associated with reduced adiposity and impaired adipocyte differentiation mediated by platelet-derived growth factor receptor α (PDGFRα)-dependent remodeling of preadipocytes [4]. Interestingly, we also showed that Nck1 deficiency leads to increased expression of the specific PDGFRα ligand, PDGF-A, suggesting that this may contribute to enhanced activation of PDGFRα signaling in the absence of Nck1 in WAT. Although the exact mechanism by which Nck1 deficiency enhances PDGFRα activation remains to be elucidated, our findings are consistent with the fact that constitutive activation of PDGFRα inhibits WAT development and leads to lipodystrophy in mice [5]. Therefore, further understanding the regulation of PDGFRα activation and signaling by Nck1 could reveal an interesting avenue to harness adipogenesis during abnormal development of WAT.

Nuclear factor erythroid-2-related factor 2 (Nrf2), a transcription factor regulating the expression of antioxidant proteins in response to oxidative stress, inflammation, and apoptosis [6], has been involved in adipocyte differentiation [7, 8]. However, both Nrf2 deletion and activation impair adipogenesis, suggesting that Nrf2-mediated regulation of adipogenesis is complex (reviewed in [9]). Interestingly, Nrf2 promotes PDGF-A gene transcription by recruiting specificity protein 1 (Sp1) to the PDGF-A gene promoter during the progression of Hepatocellular carcinoma [10]. Given we previously showed that Nck1 deficiency in pancreatic β cells enhances Nrf2 activity [11], we postulate that Nrf2 activation drives enhanced PDGF-A gene transcription to promote PDGFRα activation and signaling associated with impaired adipocyte differentiation upon Nck1 deficiency.

Herein, similar to our previous findings using stromal vascular fraction (SVF) derived from WAT [4], we show that Nck1 is required for adipocyte differentiation of primary bone marrow mesenchymal stem/stromal cells (BM-MSCs) as well as C3H10T1/2 mesenchymal stem cells. In addition, we demonstrate that *Nck1*^*−/−*^ BM-MSCs display increased PDGFRα signaling that correlates with enhanced gene transcription of the PDGF ligands, PDGF-A, and -C. We also report that the activation of the PDGFRα-Nrf2 pathway linked to increased expression of PDGF-A in *Nck1*^*−/−*^ BM-MSCs. Further supporting a role for Nrf2 in mediating the effects of Nck1 deficiency in mesenchymal stem cells, we show that pharmacological inhibition of Nrf2 rescues adipogenesis in *Nck1*^*−/−*^ BM-MSCs and Nck1-depleted C3H10T1/2 mesenchymal stem cells.

## Materials and methods

### Animal studies

*Nck1*^*−/−*^ mice were initially generated in Dr. T. Pawson’s laboratory (Toronto, ON, Canada) as previously described [12]. Offspring mice, including *Nck1*^*+/+*^ mice as control littermates and *Nck1*^*−/−*^ mice, were derived from heterozygous *Nck1*^+/−^ mating pairs previously provided by Dr. Nina Jones (Guelph University, Guelph, ON, Canada). Throughout the study, mice were kept in an animal room maintained at 21 °C with fixed 12:12-h dark-light cycles and free access to food and water. Male mice have been used in all experiments and the McGill University Animal Care Committee approved the mice handling procedures (protocol #7601).

### Cell lines

Primary BM-MSCs were essentially isolated from mice at 4–6 or 16–20 weeks post-weaning according to previously reported procedures [13] and cultured in αMEM with 10% FBS at 37 °C in a humidified atmosphere containing 5% CO_2_ incubator for 24 h. Then, the cells were washed, replenished with fresh media and cultured for 5 days. The initial spindle-shaped cells appear after 3 days and the cells reach up to 80–90% confluency by day 5 of culture. The cells are then harvested using 0.25% trypsin twice and plated at specific density before to be used for experiments (passage 3). C3H10T1/2 mesenchymal stem cells (ATCC) were cultured in αMEM with 10% heat-inactivated Fetal Bovine Serum (FBS) at 37 °C and 5% CO_2_.

### In vitro adipocyte differentiation

Both BM-MSCs and C3H10T1/2 cells were cultured until confluency and then induced for adipocyte differentiation in αMEM with 10% FBS supplemented with 1 μmol/L dexamethasone, 5 μg/mL rosiglitazone, 0.5 mmol/L 3-isobutyl-1-methylxanthine (IBMX), and 3 μg/mL (500 nM) insulin for 3 days and then maintained in the same medium without IBMX until the indicated time point for harvest. Lipid droplet formation was visualized using light microscopy, and Oil red O (ORO) staining. For ORO staining, cells were fixed in 10% PBS-buffered formalin for 15 min, permeabilized using 60% isopropanol for 5 min, and stained with 0.18% ORO for 15 min. Upon washing, ORO was eluted in 100% isopropanol for 10 min and absorbance was quantified at 492 nm.

### In vitro osteogenic differentiation

Both BM-MSCs and C3H10T1/2 cells were cultured until confluency and then induced for osteoblast differentiation in αMEM with 10% FBS supplemented with 50 μg/mL L-Ascorbic acid, and 10 mM β-glycerophosphate. Osteoblast differentiation media was replenished every other day for 10 days. Alizarin Red S (AZR) and alkaline phosphatase (ALP) staining were performed to assess osteogenic differentiation. Briefly, 2 g of AZR powder was dissolved in 100 mL water (PH 4.1–4.3). Fixed cells were incubated with the AZR solution for 45 min at room temperature. Calcium deposition is shown as a red stain. For the ALP staining, fixed cells were incubated with BCIP/NBT substrate solution for 10 min at room temperature. Alkaline phosphate is shown as a purple stain. At the end of differentiation, total RNA was extracted in parallel wells to assess osteogenic gene expression as reported below.

### siRNA transfection and cell treatments

C3H10T1/2 mesenchymal stem cells were reverse transfected with 10 nmol/L of control or Nck1 (Mouse) siRNA duplex (sequence 1: rGrCrArGrUrUrGrUrCrArArUrArArCrCrUrArArArUrArCGG, sequence 2: rCrCrcGrUrArUrUrUrArGrGrUrUrArUrUrGrArCrArArCrUrGrC) (IDT) using Lipofectamine RNAiMAX Reagent (Invitrogen). To activate Nrf2, indicated BM-MSCs and C3H10T1/2 cells at confluency were treated with 10 μM *tert*-butylhydroquinone (t-BHQ) dissolved in dimethyl sulfoxide (DMSO) or DMSO alone. Adipogenesis was induced after a 24 h pre-treatment with t-BHQ or DMSO using the regular differentiation cocktail supplemented with 10 μM t-BHQ or DMSO. The expression of genes was evaluated after the initial 24 h treatment and adipogenesis induction as reported below. To inhibit Nrf2, indicated BM-MSCs at confluency and C3H10T1/2 cells 48 h post siRNA transfection were treated with 10 μM the Nrf2 pharmacological inhibitor, ochratoxin A (OTA) dissolved in ethanol (EtOH), or EtOH alone. Adipogenesis was induced after a 24 h pre-treatment with OTA or EtOH using the regular differentiation cocktail supplemented with 10 μM OTA or EtOH. As reported above, the expression of genes was evaluated after the initial 24 h treatment and adipogenesis induction.

### Western blotting

Equal amounts of triton soluble cellular proteins (10–30 μg) were resolved by SDS-PAGE and transferred to polyvinylidene fluoride (PVDF) membrane (Bio-Rad). Following incubation in Tris-buffered saline containing 0.01% Tween-20 (TBS-T) and 10% dry milk or 5% BSA, membranes were probed with the following antibodies from Cell Signaling Technology: Hsp90 (4877S), Akt (9272), pAkt Ser473 (9271 L), PPARγ (2435), aP2 (3544), adiponectin (2789), and perilipin (9349). Nrf2 (sc-722) antibody was purchased from Santa Cruz Biotechnology. Membranes were then incubated with appropriate HRP-conjugated secondary antibodies. Signal was detected by chemiluminescence using the ChemiDoc Touch Imaging System (Bio-Rad) and quantified with ImageLab software (Bio-Rad).

### RNA extraction and quantitative real-time PCR

Total RNA was extracted using TRIzol reagent according to the manufacturer instructions (Invitrogen). cDNA synthesis was performed using a High-Capacity cDNA Reverse Transcription Kit according to the manufacturer (Applied Biosystems). Quantitative PCR was performed using the *PowerUp* SYBR Green PCR Master Mix (Applied Biosystems) in a ViiA 7 thermal cycler system (Applied Biosystems). Expression levels were calculated using the ∆∆Ct method normalized to the housekeeping gene Cyclophilin B or GAPDH, whose expression remained constant throughout the treatments. Specific primers for PCR amplification of targeted genes were used, and their sequences are available upon request.

### BrdU incorporation assay

Indicated BM-MSCs were incubated with BrdU (3 μg/mL) for 2 h followed by fixation and DNA denaturation. Incorporated BrdU was detected using BrdU and Alexa fluoro 594 donkey anti-mouse antibodies (Invitrogen). Signal was visualized using a confocal Zeiss microscope (LSM 510 META) and the number of BrdU positive cells was quantified through ImageJ (400–700 cells counted/group).

### Flow cytometry analysis

To assess the BM-MSCs count, cells were isolated from *Nck1*^*+/+*^ and *Nck1*^*−/−*^ mice as described above and used for flow cytometry analysis at passage 3. BM-MSCs were dissociated using a non-enzymatic dissociation buffer and resuspended in PBS/0.1%BSA. BM-MSCs were stained with the following anti-mouse antibodies: FITC CD31 **(**Clone: 390; BioLegend 102405), PerCP/Cy5.5 CD45 **(**Clone: 30-F11; BioLegend 103131), Pacific Blue Sca-1 (clone D7; BioLegend 108119), and PE CD140a (PDGFRα) **(**Clone: APA5; BioLegend 135905) for 1 h at 4 °C. The stained BM-MSCs were then sorted using a BD FACSCanto II flow cytometer. Data was quantified and analyzed using FACSDiva.

### Statistics

Data analysis was performed using unpaired Student *t-test* on Prism software (GraphPad Prism Software version 8.2.1), and *p* ≤ 0.05 was considered to be significant.

## Results

### Nck1 deficiency impairs adipogenesis in mesenchymal stem cells

**Fig. 1:**
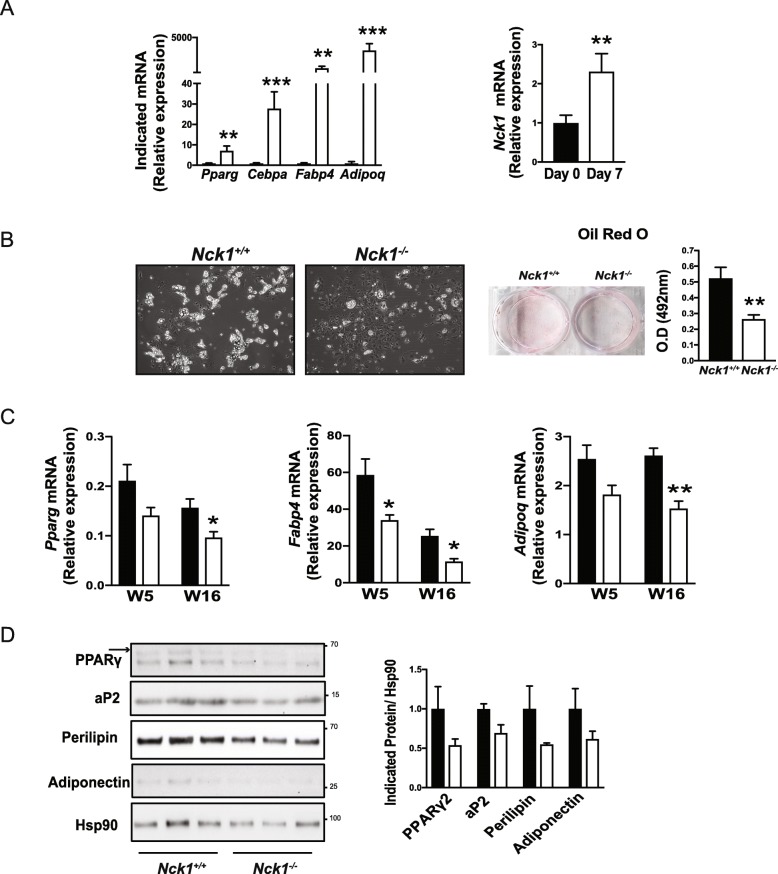
Nck1 is required for differentiation of BM-MSCs into adipocytes. **a** Adipogenesis markers and Nck1 relative mRNA levels before (Day 0, black bars) and upon induction of adipogenesis (Day 7, white bars) in *Nck1*^*+/+*^ BM-MSCs isolated from week 5 post-weaning mice (*n* = 4/group). **b** Representative images (DIC, 10X) and Oil red O staining pictures and quantification in Day 7 differentiated *Nck1*^*+/+*^ and *Nck1*^*−/−*^ BM-MSCs isolated from week 5 post-weaning mice (*n* = 4/group). **c** Relative *Pparg*, *Fabp4*, and *Adipoq* mRNA levels in day 7 differentiated *Nck1*^*+/+*^ (black bars) and *Nck1*^*−/−*^ (white bars) BM-MSCs isolated from week 5 post-weaning (W5) and week 16 post-weaning (W16) mice (*n* = 3–4/group). **d** Expression of adipogenesis markers at 7 days of differentiation as determined by western blot and densitometry relative to Hsp90 in *Nck1*^*+/+*^ (black bars) and *Nck1*^*−/−*^ (white bars) BM-MSCs isolated from week 5 post-weaning mice (*n* = 3/group). Arrow represents PPARγ2. Data are mean ± SEM. Statistical significance evaluated by unpaired Student’s t-test and is reported as *p ≤ 0.05, **p ≤ 0.01, and ***p ≤ 0.001

As expected, induction of adipocyte differentiation in primary BM-MSCs increases mRNA levels of the major adipogenesis markers, *Pparg, Cebpa,* and their downstream targets, *Fabp4,* and *Adipoq* (Fig. 1a, left panel). To confirm the multipotent nature of these BM-MSCs, we assessed their osteogenic potential. As shown in Additional file 1: Figure S1, induction of osteogenic differentiation leads to changes in BM-MSCs morphology associated with phenotypic and genes characteristics of osteoblasts (Additional file 1: Figure S1). To investigate a potential role of Nck1 in adipogenic differentiation in BM-MSCs, we first monitor Nck1 mRNA levels before and after induction of adipocyte differentiation. Interestingly, induction of adipocyte differentiation in primary BM-MSCs results in increased Nck1 mRNA (Fig. 1a, right panel), suggesting that Nck1 might be involved in this process. Indeed, as we previously reported in WAT derived stromal vascular fraction, and murine 3 T3-L1 and human SGBS preadipocytes [4], Nck1 deficiency in BM-MSCs and Nck1 downregulation in C3H10T1/2 cells impair adipocyte differentiation as shown by reduced accumulation of lipid droplets and Oil red O staining following induction of differentiation (Fig. 1b and Additional file 2: Figure S2A). Supporting this, *Pparg, Fabp4,* and *Adipoq* levels are significantly reduced in *Nck1*^*−/−*^ BM-MSCs isolated from younger (week 5 post-weaning) and older (week 16 post-weaning) mice (Fig. 1c). Nck1 depleted C3H10T1/2 mesenchymal stem cells also show a tendency toward reduced level of these markers before differentiation and to a lower extend upon differentiation (Additional file 2: Figure S2B). In agreement, western blot analysis at day 7 of differentiation demonstrates a tendency toward reduced protein levels of the main adipocyte differentiation markers PPARγ2, aP2, perilipin, and adiponectin in *Nck1*^*−/−*^ BM-MSCs (Fig. 1d). Reduced adipocyte differentiation in *Nck1*^*−/−*^ BM-MSCs cannot be attributed to a fewer number of precursors cells because the number of Lin- cells and Lin-;Sca1+;PDGFRα+ precursors are comparable in P3 BM-MSCs derived from *Nck1*^*+/+*^ and *Nck1*^*−/−*^ mice (Additional file 3: Figure S3). Therefore, these results provide strong evidence that Nck1 is also required for mesenchymal stem cells differentiation into adipocyte.

### Nck1 deficiency promotes the expression of PDGF ligands and PDGFRα signaling

**Fig. 2:**
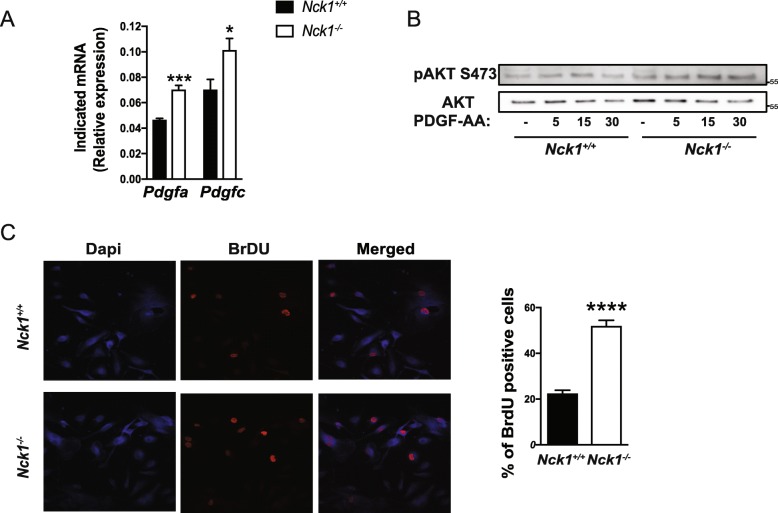
Nck1 deficiency in BM-MSCs promotes expression of PDGF-A and -C, PDGFRα signaling, and proliferation. **a** Relative *Pdgfa* and *Pdgfc* mRNA levels in growing *Nck1*^*+/+*^ and *Nck1*^*−/−*^ BM-MSCs isolated from week 5 post-weaning mice (*n* = 4/group). **b** PDGF-AA (25 ng/mL)-induced pAKT in over-night starved *Nck1*^*+/+*^ and *Nck1*^*−/−*^ BM-MSCs isolated from week 5 post-weaning mice (representative of 3 independent experiments). **c** BrdU incorporation and quantification of BrdU positive *Nck1*^*+/+*^ and *Nck1*^*−/−*^ BM-MSCs isolated from week 5 post-weaning mice (*n* = 4/group). Data are mean ± SEM. Statistical significance evaluated by unpaired Student’s t-test and is reported as *p ≤ 0.05, ***p ≤ 0.001, and ****p ≤ 0.0001

As we previously observed in Nck1 depleted 3 T3-L1 preadipocytes, *Nck1*^*−/−*^ BM-MSCs and Nck1 depleted C3H10T1/2 mesenchymal stem cells also show higher mRNA levels of the PDGF ligands, PDGF-A and -C (Fig. 2a, and Additional file 2: Figure S2C), whereas the expression of other PDGF ligands, PDGF-B and D, is below the detectable level. In addition, PDGF-AA leads to higher induction of Akt phosphorylation in *Nck1*^*−/−*^ compared to *Nck1*^*+/+*^ BM-MSCs (Fig. 2b), suggesting enhanced PDGFRα signaling following Nck1 deficiency. In agreement with the important role of PDGF ligands in promoting PDGFRα signaling and proliferation in mesenchymal stem cells (as reviewed in [14]), we demonstrate that the higher expression of PDGF ligands and increased PDGFRα signaling in *Nck1*^*−/−*^ BM-MSCs also correlate with enhanced proliferation as revealed by increased number of BrDU positive cells in *Nck1*^*−/−*^ BM-MSCs compared to *Nck1*^*+/+*^ BM-MSCs (Fig. 2c).

### Nck1 deletion promotes Nrf2 activation

**Fig. 3:**
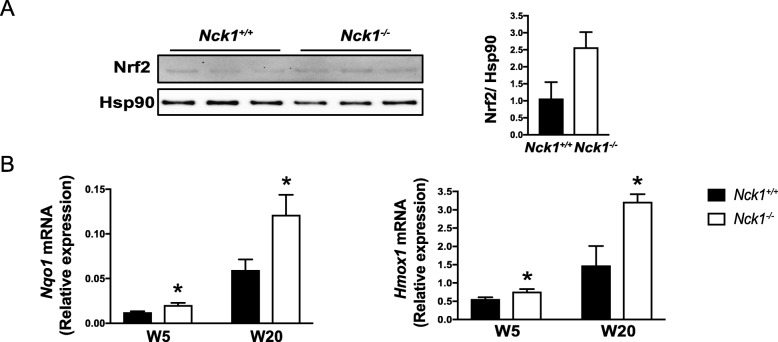
Nck1 deletion leads to Nrf2 activation in BM-MSCs. **a** Western blots of Nrf2 and quantification relative to Hsp90 in growing *Nck1*^*+/+*^ and *Nck1*^*−/−*^ BM-MSCs isolated from week 5 post-weaning mice (*n* = 3/group). **b** Relative *Nqo1* and *Hmox1* mRNA levels in growing *Nck1*^*+/+*^ and *Nck1*^*−/−*^ BM-MSCs isolated from week 5 post-weaning (W5) and week 20 post-weaning (W20) mice (*n* = 3–4/group). Data are mean ± SEM. Statistical significance evaluated by unpaired Student’s t-test and is reported as *p ≤ 0.05

*Nck1*^*−/−*^ BM-MSCs display increased Nrf2 protein levels compared to *Nck1*^*+/+*^ BM-MSCs (Fig. 3a). This correlates with increased mRNA expression of the Nrf2 target genes, *Nqo1 and Hmox1,* in *Nck1*^*−/−*^ BM-MSCs (Fig. 3b), demonstrating enhanced Nrf2 activation in Nck1-deficient BM-MSCs. Interestingly, although enhanced activation of Nrf2 is already observed in BM-MSCs isolated from 5 weeks old mice (post-weaning), this is accentuated in BM-MSCs isolated from older mice (20 weeks post-weaning) and inversely correlates with the ability of Nck1-deficient BM-MSCs to differentiate into adipocyte (Fig. 1c). Collectively, these results show that Nck1 deficiency in mesenchymal stem cells, while preventing adipocyte differentiation, promotes expression of specific PDGF ligands, PDGFRα signaling, and Nrf2 activation.

### Nrf2 activation is dependent on PDGFRα activation

**Fig. 4:**
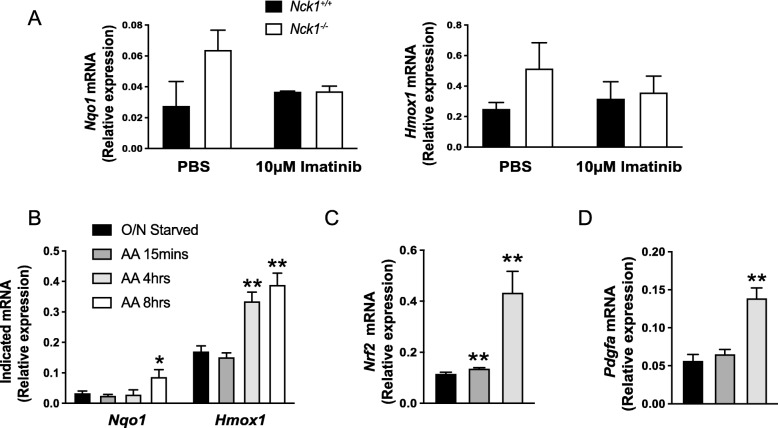
Activation of Nrf2 is dependent on PDGFRα. **a** Relative *Nqo1* and *Hmox1* mRNA levels in Imatinib (10 μM, 24 h) or PBS treated growing *Nck1*^*+/+*^ and *Nck1*^*−/−*^ BM-MSCs isolated from week 5 post-weaning mice (n = 3–5/group). **b** Relative *Nqo1* and *Hmox1* mRNA levels in PDGF-AA (25 ng/mL)-stimulated (15 min, 4 and 8 h) over-night starved *Nck1*^*+/+*^ BM-MSCs isolated from week 5 post-weaning mice (*n* = 3–4/group). Relative *Nrf2* (**c**) and *Pdgfa* (**d**) mRNA levels in PDGF-AA (25 ng/mL)-stimulated (15 min and 4 h) over-night starved *Nck1*^*+/+*^ BM-MSCs isolated from week 5 post-weaning mice (*n* = 4/group). Data are mean ± SEM. Statistical significance evaluated by unpaired Student’s t-test and is reported as *p ≤ 0.05, and **p ≤ 0.01

Enhanced induction of Nrf2 target genes (*Nqo1 and Hmox1*) in *Nck1*^*−/−*^ BM-BSCs is prevented by overnight treatment with the PDGFR kinase inhibitor, Imatinib (Fig. 4a), suggesting that Nrf2 activation is dependent on PDGFR activity. To demonstrate that Nrf2 activation is related to PDGFRα activation and signaling, serum overnight-starved *Nck1*^*+/+*^ BM-MSCs were stimulated with PDGF-AA to specifically activate PDGFRα and downstream signaling. Interestingly, PDGF-AA long-term stimulation for 4 and 8 h leads to a significant increase in the mRNA levels of Nrf2 target genes (*Nqo1 and Hmox1*) and also Nrf2 mRNA levels (Fig. 4b and c), suggesting that Nrf2 expression and activation belongs to PDGFRα signaling. We previously demonstrated that the induction of PDGF-A mRNA is PDGFRα-dependent in Nck1 depleted 3 T3-L1 preadipocytes [4]. In agreement, we found that PDGF-AA stimulation induces higher expression of *Pdgfa* in *Nck1*^*+/+*^ BM-MSCs (Fig. 4d), suggesting that increased transcription of the PDGF-A gene in Nck1-deficient BM-MSCs is dependent on PDGFRα activation and signaling.

### Activation of Nrf2 mimics Nck1 deficiency

**Fig. 5:**
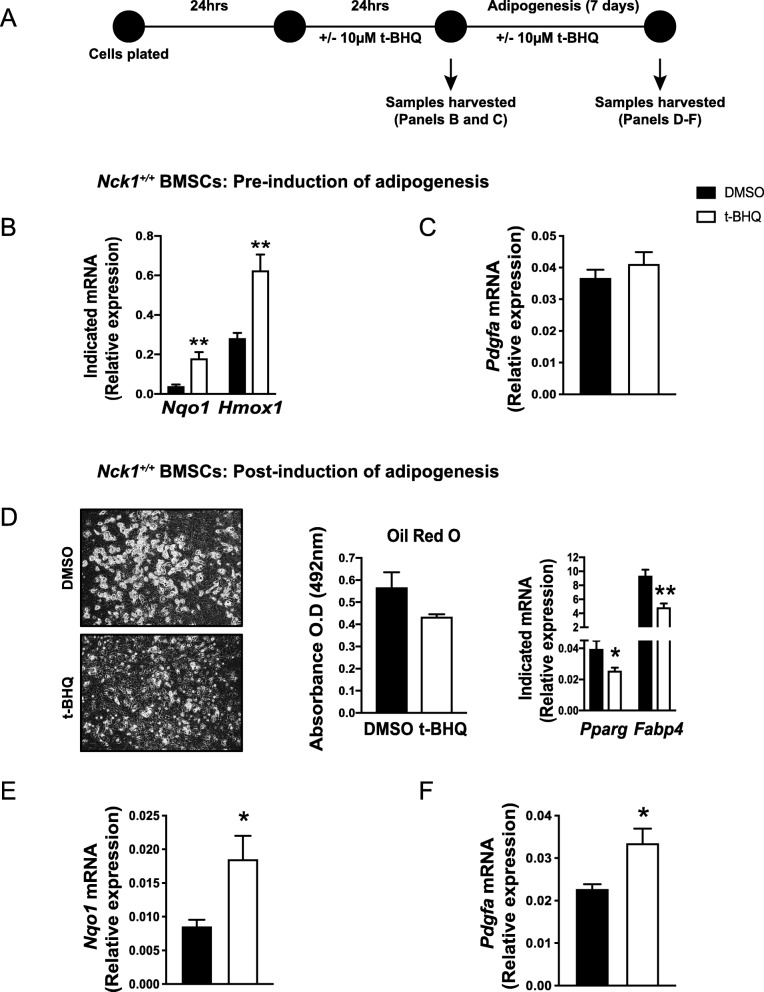
Activation of Nrf2 induces PDGF-A expression and impairs adipogenesis in BM-MSCs**. a** Experimental design. Relative *Nqo1* and *Hmox1* (**b**) and *Pdgfa* (**c**) mRNA levels in t-BHQ (10 μM, 24 h) or DMSO treated growing *Nck1*^*+/+*^ BM-MSCs derived from week 5 post-weaning mice (*n* = 4–5/group). **d** At day 7 of differentiation, representative images (DIC, 10X), Oil red O staining quantification (*n* = 3–5/group) and relative *Pparg* and *Fabp4* mRNA levels (*n* = 4/group) in t-BHQ (10 μM) or DMSO treated *Nck1*^*+/+*^ BM-MSCs isolated from week 5 post-weaning mice. At day 7 of differentiation, relative *Nqo1* (**e**) and *Pdgfa* (**f**) mRNA levels in t-BHQ (10 μM) or DMSO treated *Nck1*^*+/+*^ BM-MSCs isolated from week 5 post-weaning mice (*n* = 4/group). Data are mean ± SEM. Statistical significance evaluated by unpaired Student’s t-test and is reported as *p ≤ 0.05, and **p ≤ 0.01

To investigate whether Nrf2 mediates the induction of PDGF-A mRNA, *Nck1*^*+/+*^ BM-MSCs were treated with t-BHQ for 24 h before the induction of adipogenesis (Fig. 5a). As expected from an activator of Nrf2, exposure of *Nck1*^*+/+*^ BM-MSCs to t-BHQ increases *Nqo1 and Hmox1* expression levels (Fig. 5b). However, t-BHQ does not affect *Pdgfa* levels (Fig. 5c). On the other hand, t-BHQ maintained during the induction of *Nck1*^*+/+*^ BM-MSCs adipocyte differentiation (Fig. 5a) strongly impairs adipogenesis as shown by a tendency to reduced lipid accumulation, Oil red O staining, while *Pparg* and *Fabp4* mRNA levels were significantly reduced (Fig. 5d). Interestingly, t-BHQ added to the differentiation cocktail significantly promotes expression of *Nqo1* (Fig. 5e), supporting increased Nrf2 activation in these conditions. More importantly, t-BHQ during differentiation also leads to significant increased *Pdgfa* mRNA levels (Fig. 5f), further suggesting that *Pdgfa* is a potential Nrf2 target gene. Although to a lower extent, we observe similar effects of t-BHQ in C3H10T1/2 mesenchymal stem cells (Additional file 4: Figure S4). Altogether, these results suggest that inhibition of adipogenesis in Nck1-deficient BM-MSCs and C3H10T1/2 mesenchymal stem cells relates to enhanced Nrf2 activation.

### Nrf2 activation mediates the effects of Nck1 deficiency on adipogenesis

**Fig. 6:**
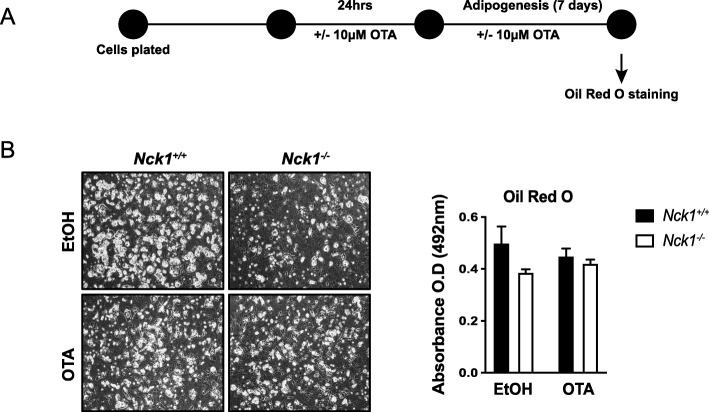
Inhibition of Nrf2 prevents the effects of Nck1 deletion on adipogenesis in BM-MSCs**. a** Experimental design. **b** At day 7 of differentiation, representative images (DIC, 10X), and Oil red O staining quantification (*n* = 3/group) in OTA (10 μM) or DMSO treated *Nck1*^*+/+*^ and *Nck1*^*−/−*^ BM-MSCs derived from week 5 post-weaning mice. Data are mean ± SEM

To further demonstrate whether impaired adipogenesis following Nck1 deficiency is dependent on Nrf2 activation, *Nck1*^*+/+*^ and *Nck1*^*−/−*^ BM-MSCs were treated with the Nrf2 inhibitor, ochratoxin A (OTA), for 24 h before the induction of adipogenesis in presence or absence of OTA (Fig. 6a). Interestingly, OTA rescues adipocyte differentiation in *Nck1*^*−/−*^ BM-MSCs (Fig. 6b) and siNck1 C3H10T1/2 mesenchymal stem cells (Additional file 5: Figure S5), further supporting that Nrf2 mediates the effects of Nck1 deficiency on adipogenesis.

## Discussion

**Fig. 7:**
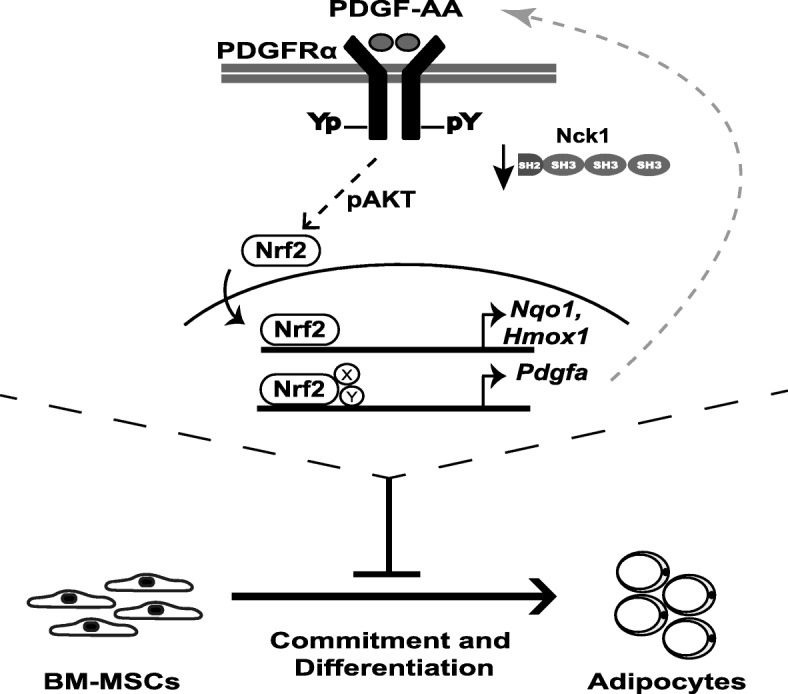
Activation of the PDGFRα-Nrf2 anti-adipogenic signaling pathway impairs adipocyte differentiation in Nck1-deficient BM-MSCs. Nck1 deficiency in BM-MSCs results in enhanced PDGFRα signaling that leads to Nrf2 activation, which upon nuclear translocation induces expression of the anti-oxidant genes, *Nqo1* and *Hmxo1*, as well as *Pdgfa* encoding PDGF-A, a specific ligand for PDGFRα. Increased PDGF-A expression could contribute to maintain higher activation of PDGFRα. Furthermore, PDGFRα-dependent Nrf2 induction leads to impaired adipocyte differentiation in Nck1 deficient BM-MSCs

Nrf2 mediated antioxidant genes expression is regulated by Nrf2 association with the Kelch-like ECH-associated protein 1 (KEAP1) [6]. Indeed, KEAP1 interaction promotes Nrf2 ubiquitination and proteasomal degradation under quiescent conditions [15]. Upon accumulation of intracellular reactive oxidative species (ROS), Nrf2 disassociates from Keap1 and is stabilized upon phosphorylation, resulting in Nrf2 nuclear translocation. Nuclear Nrf2 binds to the antioxidant-responsive element (ARE) located within the promoter of several anti-oxidative genes, including nicotinamide adenine dinucleotide phosphate (NADPH)-oxidase quinone oxidoreductase 1 (Nqo1) and heme oxygenase-1 (Hmox1) [9]. Given the implication of ROS in adipocyte differentiation [16, 17] and Nrf2 in maintaining optimal intracellular ROS levels (reviewed in [18]), a role for Nrf2 in regulating adipocyte differentiation was anticipated [7, 8]. In this perspective, our study provides evidence that activation of Nrf2 is associated with impaired adipocyte differentiation upon Nck1 deficiency. However, whether higher activation of Nrf2 in *Nck1*^*−/−*^ BM-MSCs may deplete intracellular ROS levels to below optimal levels required to support adipogenesis remains to be investigated. On the other hand, our study shows that Nck1 deficiency promotes PDGFRα signaling that leads to Nrf2 activation and Nrf2-dependent induction of PDGF-A mRNA, suggesting a potential autocrine loop involving PDGFRα-Nrf2-PDGF-A (Fig. 7). We previously demonstrated that Nck1 directly interacts with the tyrosine-phosphorylated PDGFRα through its SH2 domain and Nck1 depletion in 3 T3-L1 preadipocytes promotes PDGFRα activation and signaling [4]. This concept appears to be also valid in *Nck1*^*−/−*^ BM-MSCs, but the underlying mechanism of how Nck1 depletion promotes PDGFRα signaling remains to be addressed. It is suggested that enhanced mRNA level of PDGF-A, a specific ligand for PDGFRα, contributes to promoting PDGFRα signaling in *Nck1*^*−/−*^ BM-MSCs. Nevertheless, our study provides insights into the underlying molecular mechanism of how increased PDGFRα signaling plays a role in preventing adipocyte differentiation by identifying Nrf2 as an important player mediating PDGFRα signaling. Activation of Nrf2 could be a consequence of increased PDGFRα signaling in *Nck1*^*−/−*^ BM-MSCs, but it is also possible that Nck1 deficiency indirectly regulates Nrf2 phosphorylation and nuclear translocation by impacting the activation of a yet unidentified kinase that regulates Nrf2. In this perspective, Fyn, which belongs to the Src family kinases, phosphorylates Nrf2 at Tyr568 site to facilitate Nrf2 export from the nucleus and its binding to Keap1 [19]. Interestingly, Nck1 interacts with Fyn through its SH3 domain and in turn increases Fyn activation in podocytes leading to increase downstream signaling pathways required for podocyte function [20]. Therefore, Nck1 deletion may inhibit Fyn activation leading to Nrf2 nuclear retention due to its lower phosphorylation at Tyr568. Furthermore, AMP-activated kinase (AMPK) may serve as an upstream regulator of Nrf2 and promote Nrf2 nuclear accumulation by directly phosphorylating Nrf2 at Ser550 [21]. Given we previously showed that Nck1 deficiency in pancreatic β cells increases AMPK activation [11], this mechanism might be involved in regulating Nrf2 activation in *Nck1*^*−/−*^ BM-MSCs. Furthermore, we have also shown that Nck1 deficiency results in increased activation of PKR-like endoplasmic reticulum kinase (PERK), correlating with enhanced AMPK and Nrf2 activation in pancreatic β cells [11]. However, increased PERK activity was not detected in *Nck1*^*−/−*^ BM-MSCs (data not shown).

It is well known that BM-MSCs and C3H10T1/2 mesenchymal stem cells can differentiate into various other lineages, including osteoblasts, and chondrocytes [13, 22]. In addition, various studies provided evidence that improved adipocyte differentiation of BM-MSCs occurs at the expense of osteoblast differentiation in knock-down mice models [23, 24]. Therefore, it would be interesting to determine whether the effect of Nck1 deficiency is specific to adipogenesis or it also impacts the differentiation of mesenchymal stem cells towards other lineages. It is possible that the reduction of adipocyte differentiation resulting from Nck1 deficiency promotes differentiation into other lineages at the expense of adipogenesis.

## Conclusion

This study provides insight into a yet uncovered molecular mechanism involving PDGFRα-Nrf2-dependent regulation of adipocyte differentiation. Mechanically, Nck1 deficiency promotes PDGFRα signaling leading to Nrf2 activation, which contributes to inhibiting adipogenesis. Meanwhile, harnessing adipogenesis by facilitating activation of the PDGFRα-Nrf2 anti-adipogenic signaling pathway is an interesting avenue to oppose excessive WAT expansion leading to obesity.

## Supplementary information

**Additional file 1: Figure S1.** Differentiation of BM-MSCs into osteoblasts. (A) Representative images (DIC, 10X) of week 5 post-weaning *Nck1*^*+/+*^ mice derived BM-MSCs before and upon 10 days of differentiation. (B) Relative osteoblast markers *Bglap2*,* Runx2*, *Sp7*, and *Col1a1* mRNA levels before (black bars) and upon 10 days of differentiation (white bars) in BM-MSCs derived from week 5 post-weaning *Nck1*^*+/+*^ mice (W5) (*n* = 4/group). Data are mean ± SEM. Statistical significance evaluated by unpaired Student’s t-test is reported as *p ≤ 0.05, and **p ≤ 0.01.

**Additional file 2: Figure S2.** Effects of Nck1 depletion in mesenchymal stem cells. (A) Representative images (DIC, 10X) and Oil red O staining in day 5 differentiated siControl and siNck1 C3H10T1/2 cells (*n*=3/group). (B) Relative* Pparg, Fabp4, Adipoq,* and *Nck1* mRNA levels before (black bars) and at day 7 of differentiation (white bars) in siControl and siNck1 C3H10T1/2 cells (*n*=3/group). (C) Relative *Pdgfa* and *Pdgfc* mRNA levels in siControl and siNck1 C3H10T1/2 cells (*n*=5-6/group). Data are mean ± SEM. Statistical significance evaluated by unpaired Student’s t-test is reported as *p≤0.05, and **p≤0.01.

**Additional file 3: Figure S3.** Quantification of precursor cell count in P3 BM-MSCs. The number of Lin- cells relative to the total cells and Lin-;Sca1+;PDGFRɑ+ precursors relative to Lin- cells in P3 BM-MSCs derived from week 16 post-weaning (W16) *Nck1*^*+/+*^ and *Nck1*^*-/-*^ mice (*n*=3/group).

**Additional file 4: Figure S4.** Activation of Nrf2 induces PDGF-A expression and impairs adipogenesis in mesenchymal stem cells. (A) Experimental design. (B) Relative *Nqo1* and *Hmox1* mRNA levels in t-BHQ (10μM, 24hrs) or DMSO treated siControl and siNck1 C3H10T1/2 cells (*n*=3/group). (C) Relative *Pdgfa* mRNA levels in t-BHQ (10μM, 24hrs) or DMSO treated differentiated (Day 5) siControl and siNck1 C3H10T1/2 cells (*n*=3/group). (D) Representative images (DIC, 10X), Oil red O staining quantification (*n*=3/group), and relative *Pparg* and *Fabp4* mRNA levels (*n*=3/group) in t-BHQ (10μM) or DMSO treated differentiated (Day 5) siControl and siNck1 C3H10T1/2 cells. Relative *Nqo1* (E) and *Pdgfa* (F) mRNA levels in t-BHQ (10μM) or DMSO treated differentiated (Day 5) siControl and siNck1 C3H10T1/2 cells (*n*=3-4/group). Data are mean ± SEM. Statistical significance evaluated by unpaired Student’s t-test is reported as *p≤0.05.

**Additional file 5: Figure S5.** Inhibition of Nrf2 prevents the effects of Nck1 deletion on adipogenesis in mesenchymal stem cells. (A) Experimental design. (B) Representative images (DIC, 10X) and Oil red O staining quantification in OTA (10μM) or DMSO treated differentiated (Day 5) siControl and siNck1 C3H10T1/2 cells (*n*=3/group). Data are mean ± SEM. Statistical significance evaluated by unpaired Student’s t-test is reported as *p≤0.05.

## Data Availability

All data generated or analysed during this study are included in this published article and its supplementary information files.

## Abbreviations

BM-MSCs: Bone marrow mesenchymal stem/stromal cells
Nck: Non-catalytic region of tyrosine kinase
PDGF: Platelet-derived growth factor
PDGFR: Platelet-derived growth factor receptor
PERK: PKR-like endoplasmic reticulum kinase
PPARγ: Peroxisome proliferator-activated receptor gamma
SH: Src homology
siRNA: Small interfering ribonucleic acid
WAT: White adipose tissue
