# Supplementary material for: Activation of the PDGFRα-Nrf2 pathway mediates impaired adipocyte differentiation in bone marrow mesenchymal stem cells lacking Nck1
Source: Cell Commun Signal. 2020 Feb 14;18:26. doi: 10.1186/s12964-019-0506-4 (PMC7023715; doi:10.1186/s12964-019-0506-4)
Supplement: Supplementary file 2 — Additional file 1: Figure S1. Differentiation of BM-MSCs into osteoblasts. (A) Representative images (DIC, 10X) of week 5 post-weaning Nck1+/+ mice derived BM-MSCs before and upon 10 days of differentiation. (B) Relative osteoblast markers Bglap2, Runx2, Sp7, and Col1a1 mRNA levels before (black bars) and upon 10 days of differentiation (white bars) in BM-MSCs derived from week 5 post-weaning Nck1+/+ mice (W5) (n = 4/group). Data are mean ± SEM. Statistical significance evaluated by unpaired Student’s t-test is reported as *p ≤ 0.05, and **p ≤ 0.01. [file 12964_2019_506_MOESM1_ESM.pdf]

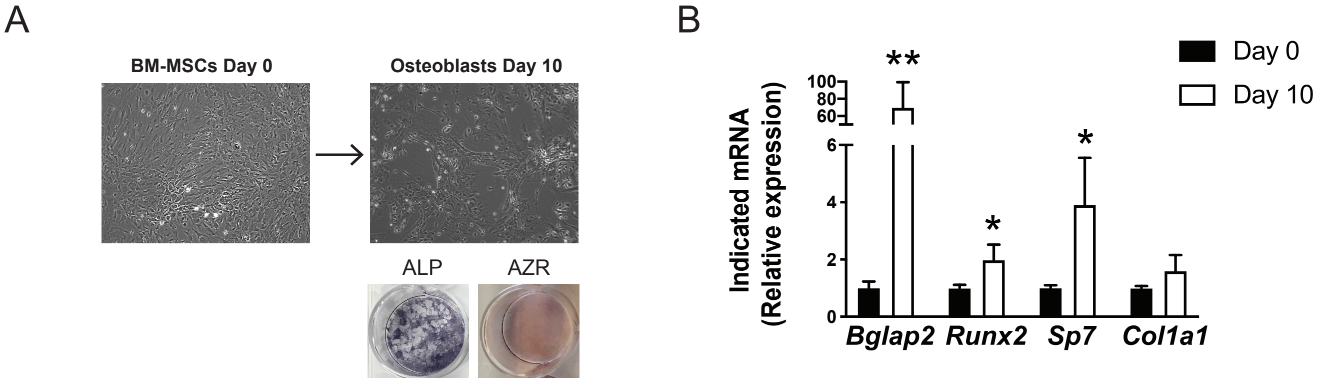

Figure S1. Differentiation of BM-MSCs into osteoblasts. (A) Representative images (DIC, 10X) of week 5 post-weaning *Nck1*$^{+/+}$ mice derived BM-MSCs before and upon 10 days of differentiation. (B) Relative osteoblast markers *Bglap2, Runx2, Sp7,* and *Col1a1* mRNA levels before (black bars) and upon 10 days of differentiation (white bars) in BM-MSCs derived from week 5 post-weaning *Nck1*$^{+/+}$ mice (W5) (n =4/group). Data are mean ± SEM. Statistical significance evaluated by unpaired Student's t-test is reported as *p≤0.05, and **p≤0.01.
