# Supplementary material for: Activation of the PDGFRα-Nrf2 pathway mediates impaired adipocyte differentiation in bone marrow mesenchymal stem cells lacking Nck1
Source: Cell Commun Signal. 2020 Feb 14;18:26. doi: 10.1186/s12964-019-0506-4 (PMC7023715; doi:10.1186/s12964-019-0506-4)
Supplement: Supplementary file 3 — Additional file 2: Figure S2. Effects of Nck1 depletion in mesenchymal stem cells. (A) Representative images (DIC, 10X) and Oil red O staining in day 5 differentiated siControl and siNck1 C3H10T1/2 cells (n=3/group). (B) Relative Pparg, Fabp4, Adipoq, and Nck1 mRNA levels before (black bars) and at day 7 of differentiation (white bars) in siControl and siNck1 C3H10T1/2 cells (n=3/group). (C) Relative Pdgfa and Pdgfc mRNA levels in siControl and siNck1 C3H10T1/2 cells (n=5-6/group). Data are mean ± SEM. Statistical significance evaluated by unpaired Student’s t-test is reported as *p≤0.05, and **p≤0.01. [file 12964_2019_506_MOESM2_ESM.pdf]

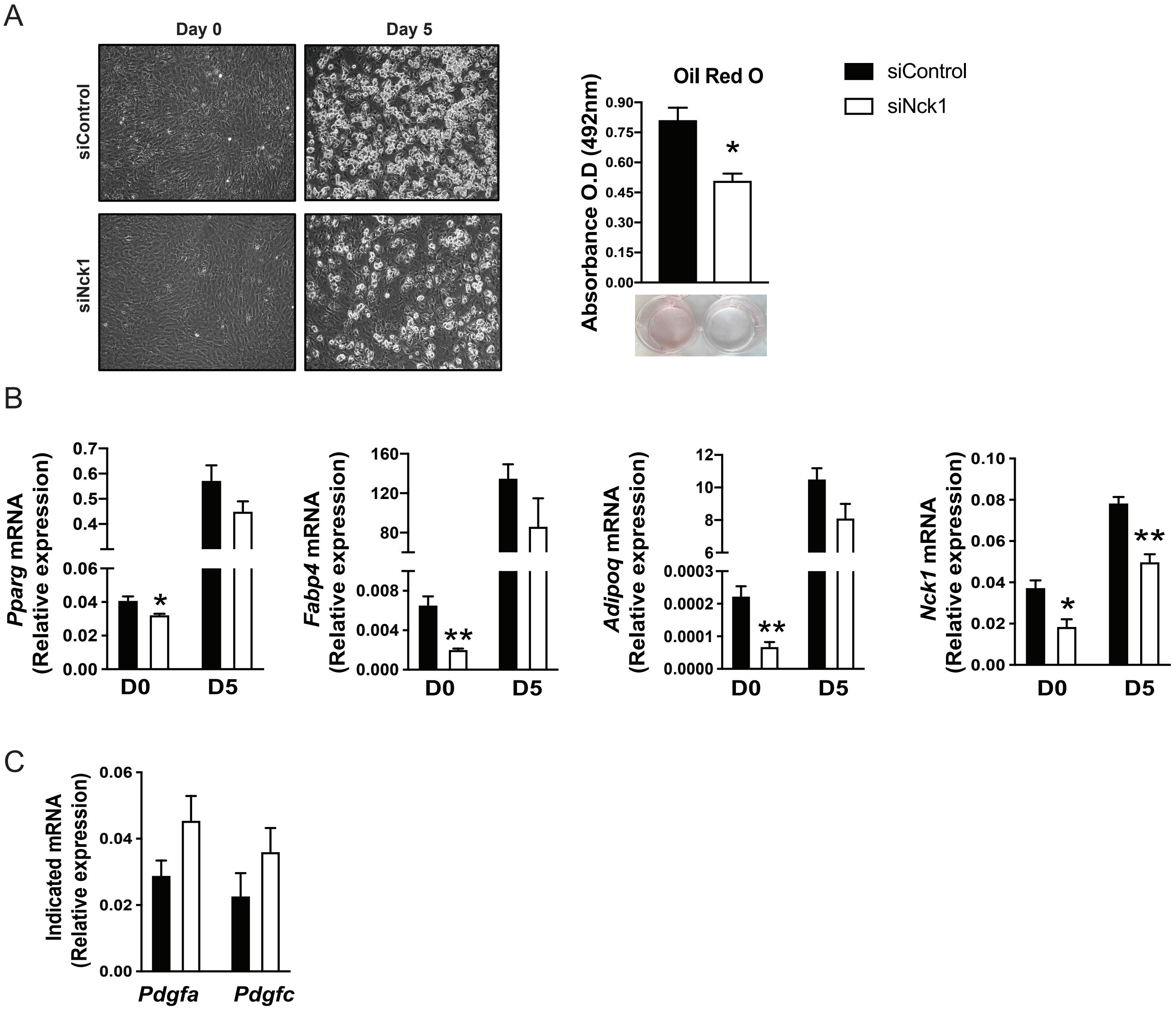


**Figure S2. Effects of Nck1 depletion in mesenchymal stem cells.** (A) Representative images (DIC, 10X) and Oil red O staining in day 5 differentiated siControl and siNck1 C3H10T1/2 cells (n=3/group). (B) Relative *Pparg, Fabp4, Adipoq,* and *Nck1* mRNA levels before (black bars) and at day 7 of differentiation (white bars) in siControl and siNck1 C3H10T1/2 cells (n=3/group). (C) Relative *Pdgfa* and *Pdgfc* mRNA levels in siControl and siNck1 C3H10T1/2 cells (n=5-6/group). Data are mean ± SEM. Statistical significance evaluated by unpaired Student's t-test is reported as *p≤0.05, and **p≤0.01.
