# Supplementary material for: Activation of the PDGFRα-Nrf2 pathway mediates impaired adipocyte differentiation in bone marrow mesenchymal stem cells lacking Nck1
Source: Cell Commun Signal. 2020 Feb 14;18:26. doi: 10.1186/s12964-019-0506-4 (PMC7023715; doi:10.1186/s12964-019-0506-4)
Supplement: Supplementary file 4 — Additional file 3: Figure S3. Quantification of precursor cell count in P3 BM-MSCs. The number of Lin- cells relative to the total cells and Lin-;Sca1+;PDGFRɑ+ precursors relative to Lin- cells in P3 BM-MSCs derived from week 16 post-weaning (W16) Nck1+/+ and Nck1-/- mice (n=3/group). [file 12964_2019_506_MOESM3_ESM.pdf]

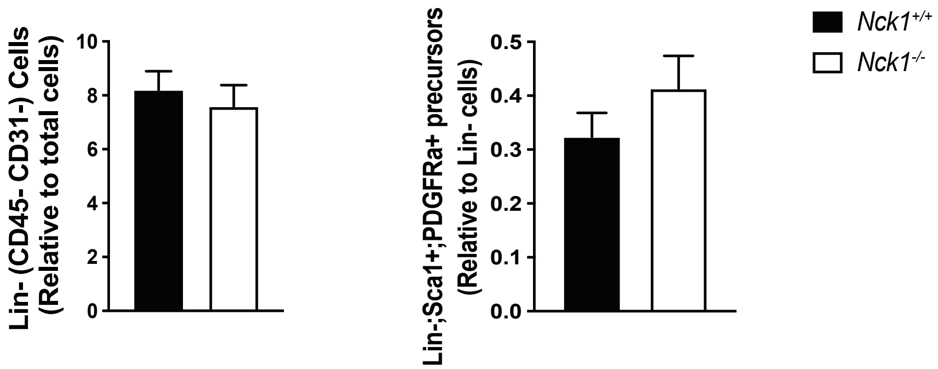

Figure S3. Quantification of precursor cell count in P3 BM-MSCs. The number of Lin- cells relative to the total cells and Lin-;Sca1+;PDGFRα+ precursors relative to Lin- cells in P3 BM-MSCs derived from week 16 post-weaning (W16) *Nck1*$^{+/+}$ and *Nck1*$^{-/-}$ mice (n=3/group).
