# Supplementary material for: Activation of the PDGFRα-Nrf2 pathway mediates impaired adipocyte differentiation in bone marrow mesenchymal stem cells lacking Nck1
Source: Cell Commun Signal. 2020 Feb 14;18:26. doi: 10.1186/s12964-019-0506-4 (PMC7023715; doi:10.1186/s12964-019-0506-4)
Supplement: Supplementary file 5 — Additional file 4: Figure S4. Activation of Nrf2 induces PDGF-A expression and impairs adipogenesis in mesenchymal stem cells. (A) Experimental design. (B) Relative Nqo1 and Hmox1 mRNA levels in t-BHQ (10μM, 24hrs) or DMSO treated siControl and siNck1 C3H10T1/2 cells (n=3/group). (C) Relative Pdgfa mRNA levels in t-BHQ (10μM, 24hrs) or DMSO treated differentiated (Day 5) siControl and siNck1 C3H10T1/2 cells (n=3/group). (D) Representative images (DIC, 10X), Oil red O staining quantification (n=3/group), and relative Pparg and Fabp4 mRNA levels (n=3/group) in t-BHQ (10μM) or DMSO treated differentiated (Day 5) siControl and siNck1 C3H10T1/2 cells. Relative Nqo1 (E) and Pdgfa (F) mRNA levels in t-BHQ (10μM) or DMSO treated differentiated (Day 5) siControl and siNck1 C3H10T1/2 cells (n=3-4/group). Data are mean ± SEM. Statistical significance evaluated by unpaired Student’s t-test is reported as *p≤0.05. [file 12964_2019_506_MOESM4_ESM.pdf]

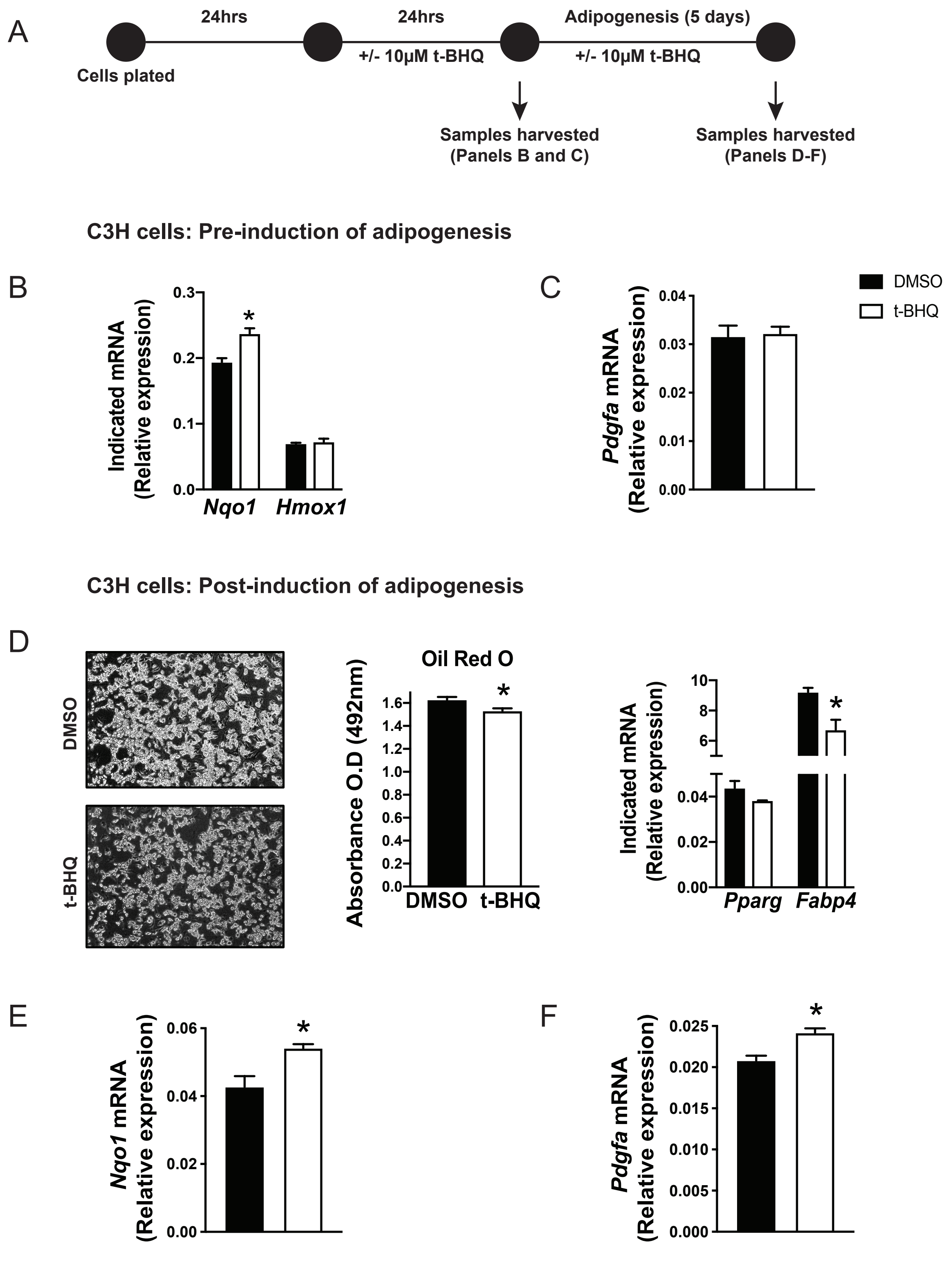

Figure S4. Activation of Nrf2 induces PDGF-A expression and impairs adipogenesis in mesenchymal stem cells. (A) Experimental design. (B) Relative *Nqo1* and *Hmox1* mRNA levels in t-BHQ (10μM, 24hrs) or DMSO treated siControl and siNck1 C3H10T1/2 cells (n=3/group). (C) Relative *Pdgfa* mRNA levels in t-BHQ (10μM, 24hrs) or DMSO treated differentiated (Day 5) siControl and siNck1 C3H10T1/2 cells (n=3/group). (D) Representative images (DIC, 10X), Oil red O staining quantification (n=3/group), and relative *Pparg* and *Fabp4* mRNA levels (n=3/group) in t-BHQ (10μM) or DMSO treated differentiated (Day 5) siControl and siNck1 C3H10T1/2 cells. Relative *Nqo1* (E) and *Pdgfa* (F) mRNA levels in t-BHQ (10μM) or DMSO treated differentiated (Day 5) siControl and siNck1 C3H10T1/2 cells (n=3-4/group). Data are mean ± SEM. Statistical significance evaluated by unpaired Student's t-test is reported as *p≤0.05.
