# Supplementary material for: Activation of the PDGFRα-Nrf2 pathway mediates impaired adipocyte differentiation in bone marrow mesenchymal stem cells lacking Nck1
Source: Cell Commun Signal. 2020 Feb 14;18:26. doi: 10.1186/s12964-019-0506-4 (PMC7023715; doi:10.1186/s12964-019-0506-4)
Supplement: Supplementary file 6 — Additional file 5: Figure S5. Inhibition of Nrf2 prevents the effects of Nck1 deletion on adipogenesis in mesenchymal stem cells. (A) Experimental design. (B) Representative images (DIC, 10X) and Oil red O staining quantification in OTA (10μM) or DMSO treated differentiated (Day 5) siControl and siNck1 C3H10T1/2 cells (n=3/group). Data are mean ± SEM. Statistical significance evaluated by unpaired Student’s t-test is reported as *p≤0.05. [file 12964_2019_506_MOESM5_ESM.pdf]

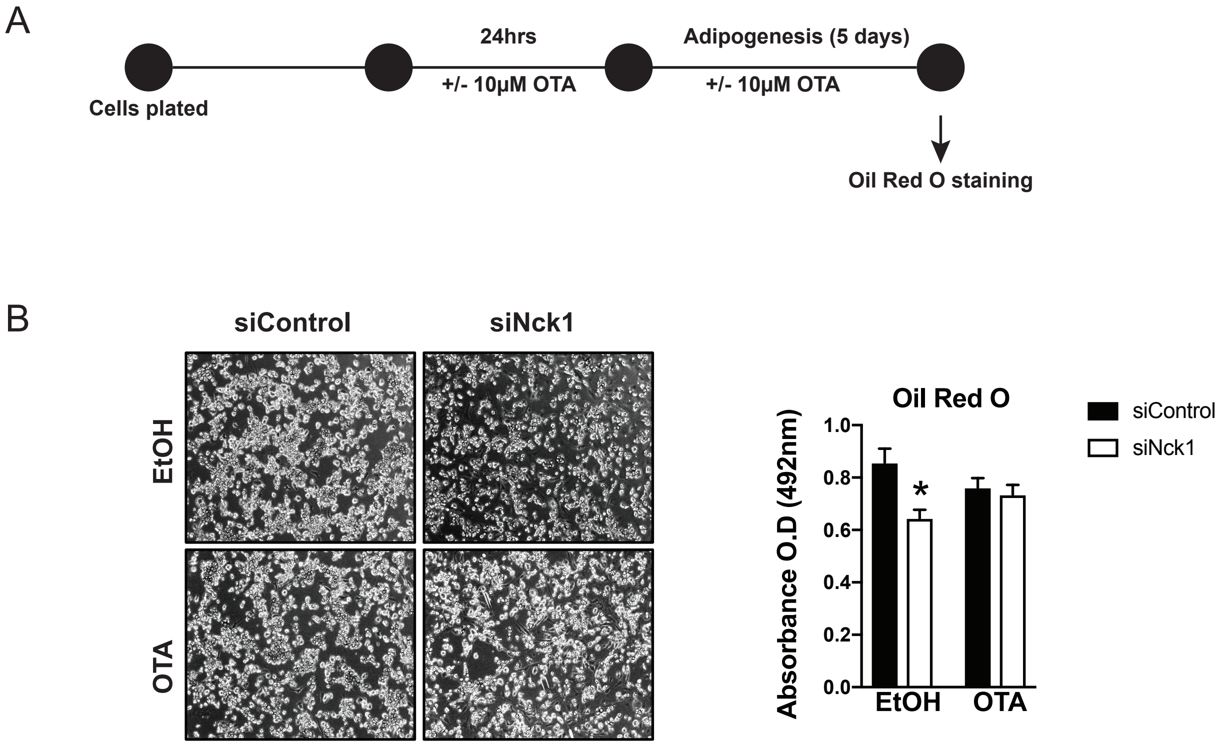

Figure S5. Inhibition of Nrf2 prevents the effects of Nck1 deletion on adipogenesis in mesenchymal stem cells. (A) Experimental design. (B) Representative images (DIC, 10X) and Oil red O staining quantification in OTA (10μM) or DMSO treated differentiated (Day 5) siControl and siNck1 C3H10T1/2 cells (n=3/group). Data are mean ± SEM. Statistical significance evaluated by unpaired Student's t-test is reported as *p≤0.05.
